# Quality of life in patients with peripheral artery disease

**DOI:** 10.1590/1677-5449.009017

**Published:** 2018

**Authors:** José Aderval Aragão, Rosely Mota Santos, Osmar Max Gonçalves Neves, Iapunira Catarina Sant’Anna Aragão, Felipe Matheus Sant’Anna Aragão, Maria Izabel Aragão Mota, Rebeca de Souza Mariano Bastos, Francisco Prado Reis

**Affiliations:** 1 Universidade Federal de Sergipe – UFS, Aracaju, SE, Brasil.; 2 Universidade Tiradentes – UNIT, Aracaju, SE, Brasil.; 3 Centro Universitário de Volta Redonda – UNIFOA, Volta Redonda, RJ, Brasil.

**Keywords:** quality of life, vascular diseases, chronic disease: peripheral arterial disease

## Abstract

**Background:**

Vascular diseases have a direct influence on quality of life (QoL) and directly affect patients’ biopsychosocial aspects. Quality of life is therefore an important element for evaluation of vascular interventions.

**Objective:**

To assess QoL in inpatients with peripheral arterial disease at a vascular surgery service in a charitable tertiary hospital.

**Methods:**

This is an exploratory study, with a cross-sectional design, conducted at a vascular surgery service in a charitable tertiary hospital, assessing patients with peripheral arterial disease using two questionnaires, one on quality of life (the WHOQOL-Bref short form) and the other on sociodemographic conditions.

**Results:**

It was observed that the physical domain, environment domain and total QoL scores were the lowest for the whole sample of 127 interviewees. Additionally, an intragroup analysis showed that men scored higher in all domains when compared with women, with the exception of the social relationships domain.

**Conclusions:**

Women with peripheral arterial disease exhibited lower scores than men in all domains of the QoL questionnaire, except for social relationships.

## INTRODUCTION

 Quality of life (QoL) is a measure that is becoming ever more important for evaluation of the negative impact on daily life of interventions and of vascular diseases such as peripheral arterial disease (PAD), i.e. of a variety of conditions that can affect people’s lives. 1 As such, QoL has been understood on the basis of physical, psychological, and social aspects, in addition to the person’s perception of their own general QoL. 2

 Under the influence of the World Health Organization (WHO) concept of health, QoL analysis has been implemented as a means of assessing states of health and wellbeing since the 1960s. 3 Since then, the study of QoL indices has become an important tool in clinical practice because of its capacity to guide better intervention strategies and aid assessment of the impact of a disease on a person’s biopsychosocial aspect. 4

 Within this context, non-transmissible chronic diseases (NTCD), especially those of vascular origin, have acquired great notoriety, even though infectious and parasitic diseases are still very prevalent in Brazil. This has happened because of the fact that NTCD have become a greater health problem in the country, responsible for 72% of the causes of death in Brazil, with diseases of the circulatory apparatus (31.3%), cancer (16.3%), diabetes mellitus (DM) (5.2%) and chronic respiratory diseases (5.8%) the leading causes. 5

 According to Pereira et al., 6 vascular diseases have a direct influence on people’s QoL because of comorbidities that cause physical deterioration and dependence on medications and interfere with daily activities, directly affecting the biopsychosocial aspect of patients. 

 Vascular diseases, and PAD in particular, alongside other NTCD, have been widely debated in the search for strategies to reduce the risk factors involved in their genesis, such as smoking bans, promotion of regular supervised physical activity, and control of glucose and lipid levels, which can be directly related to changes in people’s lifestyles and QoL. 7

 Van Hattum et al. 8 report that health-related QoL scores, especially those in the physical domain, reduced over time in patients with PAD, even after arterial bypass surgery, when paired for age and sex with members of the general population. Öztürk et al. 9 used the World Health Organization Quality of Life Instrument-Short Form (WHOQOL-Bref) to determine QoL in PAD patients after arterial bypass surgery. They detected improved QoL in the physical domain 2 weeks after femoropopliteal bypass surgery. 

 Donker et al. 10 reported that although the number of arterial bypass surgeries in PAD cases has significantly increased QoL from 2 weeks to 3 months after surgery, medium-term follow-up showed that scores returned to baseline, i.e. no increase in QoL is observed in the medium-term after bypass surgery. 

 The objective of this study was to assess QoL in inpatients with PAD at a vascular surgery service at a tertiary hospital in Aracaju, SE, Brazil. 

## MATERIALS AND METHODS

 This is an exploratory study, with a cross-sectional design, conducted at a vascular surgery service at a tertiary charitable hospital located in the town of Aracaju, SE, Brazil. The investigation was conducted by administering two questionnaires: a sociodemographic scale, constructed by the researchers, and the WHOQOL-Bref QoL questionnaire. 3

 All PAD inpatients admitted to the vascular surgery service at a tertiary hospital who signed a free and informed consent form were included. Patients were excluded if at the time of interview they did not wish to take part. 

 The investigation was conducted with a non-random sample of patients selected consecutively. Questionnaires were administered to all patients with PAD in the vascular surgery ward of the hospital studied, until a sample of 127 patients had been recruited. 

 Data were collected using a questionnaire with variables related to the sociodemographic sphere, such as sex, age, educational level, alcohol use, and smoking. Information was also collected on medications and whether or not participants had any chronic diseases. 

 The abbreviated WHOQOL-Bref version was used for QoL analysis. 3 This instrument was created by the WHO with the specific purpose of assessing QoL and it is composed of four domains designed to analyze physical capacity, psychological wellbeing, social relationships, and the environment in which the subject lives. Each domain is comprised of questions with responses scored from 1 to 5. The final scores for each domain were calculated using a syntax file, which considers the responses to each item in the domain, resulting in final scores ranging from 4 to 20, which can be transformed into a scale from 0 to 100. 

 The data collected were expressed as simple frequencies and percentages for categorical variables or means and standard deviations for continuous, discrete, and ordinal variables. Analysis of variance (ANOVA) and the Tukey test for multiple comparisons were used to evaluate differences between means. Spearman coefficients were calculated for correlations between continuous, ordinal, or discrete variables. The significance level was set at 5% and the software employed was R Core Team 2017. 

 The research project was submitted to the Human Research Ethics Committee at the Universidade Federal de Sergipe and approved under protocol number CAAE 48580115.2.0000.5546 and ruling number nº 1.217.875. 

## RESULTS

 The study sample comprised 127 patients, 54.3% of whom were male. Mean age was 66 years. There was a predominance of people from provincial areas of the state of Sergipe, who were married or in stable relationships, Catholics, and retired, and who had low educational level and incomes less than or equal to the monthly minimum wage ( Table 1 ). 

**Table 1:** Socioeconomic characteristics.

**Variables**	**n** (%)
**Sex**	
Female	58 (45.7%)
Male	69 (54.3%)
**Origin**	
State capital	44 (34.6%)
Upstate	83 (65.4%)
**Marital status**	
Has partner	65 (51.2%)
Single	62 (48.8%)
**Religion**	
Catholic	110 (86.6%)
Protestant	17 (13.4%)
**Educational level**	
Illiterate	33 (26%)
Primary education	81 (63.8%)
Secondary education or higher	13 (10.2%)
**Employment status**	
Working	25 (19.7%)
Retired	102 (80.3%)
**Family income**	
0 to 1 times minimum wage	93 (73.2%)
2 times minimum wage or greater	34 (26.8%)

 The majority of the sample, 101 individuals (79.5%), stated they had attended a consultation with a physician on the Family Health Program during the previous 12 months. Ninety-five (74.8%) of the interviewees stated they did not smoke or drink. Additionally, 92 (72.4%) of the interviewees had had surgery, such as cholecystectomy, inguinal hernia repair, hysterectomy, procedures to treat varicose veins of the lower limbs (LL), vasectomy, prostatectomy, and cataract surgery, and 59 (46.5%) had had an LL amputation. The majority of participants also reported LL pain and 77 (60.6%) were unable to walk. 

 The most prevalent associated comorbidities in the study sample were systemic arterial hypertension (SAH) and DM. Table 2 lists the frequencies of comorbidities in the sample and the medications used to treat them. 

**Table 2:** Prevalent associated comorbidities and most common medications used.

**Comorbidities**	**n** (%)
**Systemic arterial hypertension**	99 (78.0%)
Angiotensin-converting enzyme inhibitors	53 (41.7%)
Angiotensin II receptor blockers	58 (45.7%)
Diuretics	18 (14.2%)
Calcium channel blockers	10 (7.9%)
Beta blockers	9 (7.1%)
Alpha 2-agonists	3 (2.4%)
**Diabetes mellitus**	27 (21.4%)
Metformin	42 (33.1%)
Sulfonylurea	51 (40.2%)
NPH Insulin	3 (2.4%)
DPP4 inhibitor (vildagliptin)	3 (3.1%)

 Of the overall means, the lowest scores were obtained on the physical domain, the environment domain, and on total QoL, with, respectively, 64.2±27.9, 97.2±20.3, and 97.9±20.4 points, while the highest score was for the social relationships domain, with a mean of 150.2±28.9 points. Additionally, there were significant variations in the physical (p = 0.024), psychological (p = 0.015), and environment (p = 0.020) domains and in total quality of life (p = 0.039), as shown in Table 3 . 

**Table 3:** QoL Scores.

**QoL domains**	**Total** **Mean±SD**	**Men** **Mean±SD**	**Women** **Mean±SD**	***p*-value**
Physical	64.2±27.9	35.0±15.3	29.2±12.6	0.024
Psychological	113.2±27.4	59.8±13.5	53.4±13.9	0.015
Social relationships	150.2±28.9	74.9±15.3	75.3±13.6	0.873
Environment	97.2±20.3	50.8±10.8	46.4±9.5	0.020
Total QoL	97.9±20.4	51.1±10.3	46.8±10.1	0.039

SD: standard deviation; QoL: quality of life.

 The intragroup analysis of QoL revealed that men had significantly higher mean scores than women in all domains (p = 0.098) except the social relationships domain, in which women had higher scores (p = 0.873). 

 With regard to sociodemographic variables, people with a higher educational level (secondary education or higher) and people who were working had significantly higher QoL scores than people with low educational level (p = 0.26) and people who were not working (p < 0.05) in all domains except social relationships. 

 Patients who reported regular physical activity and pain in the LL exhibited better mean scores on the physical (p=0.27) and environment domains (p = 0.28), except social relationships, when compared with those who do not practice physical activities. Additionally, interviewees who reported that LL pain was an impediment to walking had lower scores in all domains (p = 0.01). It is notable that presence of wounds had a negative impact on the physical (p = 0.011) and psychological domains (p = 0.013) and on total QoL scores (p = 0.003). 

 Analysis of the most prevalent associated comorbidities showed that only SAH had a negative effect on QoL, in the psychological and environment domains and on total QoL (p = 0.009; p = 0.022; and p = 0.008, respectively). No interference in QoL by type of medication used could be detected because of the large variety of associations. 

## DISCUSSION

 The increasing numbers of people with NTCD, and PAD in particular, throughout Brazil is a concern for the Ministry of Health. 5 This is the reality of life for many people and it has been observed that they have low scores in all QoL domains except for social relationships, as was found in this study. 

 The findings of this study showed that people with PAD who were male and had higher educational level had better QoL. These findings are in line with what has been described by Carvalho et al., 11 who studied the influence of SAH on QoL. 

 With relation to gender, Ahluwalia et al. 12 ^,^ 13 and Nogales 14 state that the lower QoL scores for women than for men are possibly associated with factors such as biological differences and new roles in society and also with increased life expectancy. With regard to educational level, Saraiva et al. 15 suggest that greater comprehension of information may enable patients to understand the disease better and, as a consequence, be more compliant with treatment. 

 Oza et al. 4 used the WHOQOL-Bref and the MINICHAL as QoL assessment instruments, finding differences in mean scores of the WHOQOL-Bref domains, showing that the social relationships domain was scored lower and the physical domain was scored higher in the WHOQOL-Bref. 

 Çeviker et al. 16 also used the WHOQOL-Bref and showed that patients with chronic venous diseases had lower QoL scores than controls and that, in a univariate analysis, all domains (physical, psychological, social relationships, and environment) were affected. These findings were partially similar to those of the present study. 

 Malaquias et al., 17 Evangelista et al., 18 and Costa et al. 19 have reported that one vascular disease, venous ulcers, can cause social isolation, embarrassment, and feelings of discrimination by society and the family, which explains the finding that the psychological aspect score is low and agrees with the low score for the psychological domain in patients with wounds. 

 According to Arslantas et al., 20 who studied SAH, inconsistency between studies of QoL may be caused by differences between the populations chosen for this type of study, by different sociodemographic characteristics, and by the severity of diseases and associated comorbidities. 

## CONCLUSIONS

 In this study, it was found that scores for the social relationships domain were high and the environment domain and total QoL scores were low. Comparison by sex showed that women had lower scores than men in all domains except social relationships. 

 In view of these results, it can be stated that this study has increased knowledge and understanding of the impact of vascular diseases on QoL. It is therefore necessary that people who have diseases in this class, health professionals, social organizations, and relatives cooperate to deal with the factors that impact negatively on these patients’ QoL. 
