# Supplementary material for: Quality of life in patients with peripheral artery disease
Source: J Vasc Bras. 2018 Apr-Jun;17(2):117–21. doi: 10.1590/1677-5449.009017 (PMC6205703; doi:10.1590/1677-5449.009017)
Supplement: Translation

# Translation: Qualidade de vida em pacientes com doença arterial periférica

**Authors:** José Aderval Aragão, Rosely Mota Santos, Osmar Max Gonçalves Neves, Iapunira Catarina Sant’Anna Aragão, Felipe Matheus Sant’Anna Aragão, Maria Izabel Aragão Mota, Rebeca de Souza Mariano Bastos, Francisco Prado Reis

**Affiliations:** 1 Universidade Federal de Sergipe – UFS, Aracaju, SE, Brasil.; 2 Universidade Tiradentes – UNIT, Aracaju, SE, Brasil.; 3 Centro Universitário de Volta Redonda – UNIFOA, Volta Redonda, RJ, Brasil.

**Keywords:** qualidade de vida, doenças vasculares, doença crônica: doença arterial periférica

## Abstract

**Contexto:**

As doenças vasculares influenciam a qualidade de vida (QV) e afetam de forma direta o aspecto biopsicossocial dos indivíduos. Sendo assim, a QV é uma importante forma de avaliação das intervenções vasculares.

**Objetivo:**

Avaliar a QV em pacientes com doença arterial periférica internados no serviço de cirurgia vascular em um hospital terciário beneficente.

**Métodos:**

Trata-se de um estudo exploratório, com desenho transversal, em um serviço de cirurgia vascular em um hospital terciário beneficente, no qual pacientes com doença arterial periférica foram avaliados através de dois questionários, sendo um a respeito de qualidade de vida (versão abreviada WHOQOL-Bref) e outro sobre as condições sociodemográficas.

**Resultados:**

Foi observado que os domínios físico, meio ambiente e QV total obtiveram os menores escores entre os 127 entrevistados. Além disso, uma análise intragrupo demonstrou que os homens obtiveram pontuação maior em todos os domínios quando comparados às mulheres, com exceção do domínio de relações sociais.

**Conclusão:**

As mulheres com doença arterial periférica apresentaram uma menor pontuação em todos os domínios do questionário de QV, exceto no de relações sociais, quando comparadas aos homens.

## INTRODUÇÃO

 A qualidade de vida (QV) é uma medida que tem se tornado cada vez mais importante para a avaliação de comprometimento da vida diária resultante de intervenções e doenças vasculares como a doença arterial periférica (DAP), ou seja, de uma variedade de condições que podem afetar a vida do indivíduo 1 . Desse modo, a QV tem sido entendida com base em aspectos físicos, psicológicos e sociais, além da percepção geral da QV do próprio indivíduo 2 . 

 Sob influência do conceito de saúde da Organização Mundial da Saúde (OMS), a análise de QV passou a ser implementada como forma de avaliação dos quadros de saúde e bem-estar desde a década de 60 3 . A partir de então, o estudo dos índices de QV se tornou uma ferramenta importante na prática clínica, por ser capaz de orientar uma melhor estratégia de intervenção e auxiliar na avaliação do impacto da doença no aspecto biopsicossocial do indivíduo 4 . 

 Dentro desse contexto, as doenças crônicas não transmissíveis (DCNT), especialmente as de origem vascular, vêm ganhando grande notoriedade, apesar de no Brasil ainda serem muito prevalentes as doenças infecciosas e parasitárias. Isso vem ocorrendo justamente devido ao fato de as DCNT constituírem o problema de saúde de maior magnitude no país, correspondendo a 72% das causas de mortes no território brasileiro, com destaque para doenças do aparelho circulatório (31,3%), câncer (16,3%), diabetes melito (DM) (5,2%) e doença respiratória crônica (5,8%) 5 . 

 De acordo com Pereira et al. 6 , as doenças vasculares influenciam de forma direta na QV dos indivíduos, devido à presença de comorbidades que implicam em prejuízo físico, dependência medicamentosa e impedimento das atividades diárias, afetando de forma direta o aspecto biopsicossocial desses indivíduos. 

 As doenças vasculares, e em especial a DAP, têm sido muito debatidas, ao lado de outras DCNT, na busca de estratégias para redução de fatores de risco envolvidos em sua gênese, como abolição do tabagismo, promoção da atividade física regular supervisionada e controle dos níveis glicêmicos e lipídicos, que podem estar diretamente relacionados às mudanças de estilo e QV dos indivíduos 7 . 

 Van Hattum et al. 8 relataram que, em pacientes portadores de DAP os escores de QV relacionada à saúde, especialmente os do domínio físico, diminuíram ao longo do tempo em pacientes portadores de DAP, mesmo após cirurgia de derivação arterial, quando pareados por idade e sexo com a população geral. Öztürk et al. 9 utilizaram o World Health Organization Quality of Life Instrument-Short Form (WHOQOL-Bref) para determinar a QV em pacientes portadores de DAP após a cirurgia de derivação arterial. Os autores encontraram melhoria na QV no domínio físico após a cirurgia de derivação fêmoro-poplítea em 2 semanas após a cirurgia. 

 Enquanto isso, Donker et al. 10 , relataram que, embora o número de cirurgias de derivação arterial nos casos de DAP tenha aumentado significativamente a QV entre 2 semanas a 3 meses após a cirurgia, o seguimento a médio prazo mostrou um retorno aos escores iniciais, ou seja, nenhum aumento de QV a médio prazo é observado após a cirurgia de derivação. 

 O presente trabalho teve por objetivo avaliar a QV em pacientes com DAP internados no Serviço de Cirurgia Vascular em um hospital terciário de Aracaju (SE). 

## MATERIAIS E MÉTODOS

 Trata-se de um estudo exploratório, com desenho transversal, em um serviço de cirurgia vascular de um hospital terciário e beneficente localizado na cidade de Aracaju. A investigação foi realizada com a aplicação de dois questionários: o sociodemográfico, confeccionado pelos pesquisadores, e o questionário de QV WHOQOL-Bref 3 . 

 Foram incluídos na pesquisa todos os indivíduos portadores de DAP internados no Serviço de Cirurgia Vascular de um hospital terciário que assinaram o Termo de Consentimento Livre e Esclarecido. Foram excluídos os indivíduos que no período da entrevista não quiseram participar da pesquisa. 

 A investigação foi realizada em uma amostra não aleatória com pacientes selecionados de forma consecutiva. Os questionários foram aplicados em todos os pacientes com DAP internados na enfermaria de cirurgia vascular do referido hospital até atingir uma amostra de 127 indivíduos. 

 Para coleta de dados foi utilizado um questionário com variáveis relativas ao âmbito sociodemográfico, como sexo, idade, escolaridade, uso de álcool e tabagismo. Foram coletadas também informações sobre uso de medicamentos e ser portador ou não de alguma doença crônica. 

 Para a análise da QV, foi utilizada a versão abreviada WHOQOL-Bref 3 . Esse instrumento foi criado pela OMS com a finalidade específica de avaliar a QV e é composto por quatro domínios que têm por objetivo analisar a capacidade física, o bem-estar psicológico, as relações sociais e o meio ambiente onde o indivíduo está inserido. Cada domínio é composto por questões cujas pontuações das respostas variam entre 1 a 5. Os escores finais de cada domínio foram calculados por uma sintaxe, que considera as respostas de cada questão que compõe o domínio, resultando em escores finais numa escala de 4 a 20, os quais podem ser transformados em escala de 0 a 100. 

 Os dados coletados foram descritos por meio de frequência simples e percentuais para as variáveis categóricas ou média e desvio padrão para as variáveis contínuas, discretas ou ordinais. Para avaliar diferenças de média, foi utilizada análise de variância (ANOVA) e teste de Tukey para múltiplas comparações. Foram calculadas correlações de Spearman para correlação entre as variáveis contínuas, ordinais ou discretas. O nível de significância adotado foi de 5% e o software utilizado foi o R Core Team 2017. 

 O projeto foi submetido ao Comitê de Ética e Pesquisa com Seres Humanos da Universidade Federal de Sergipe e aprovado sob o protocolo nº: CAAE - 48580115.2.0000.5546 e parecer nº 1.217.875. 

## RESULTADOS

 Dos 127 indivíduos que participaram da pesquisa, 54,3% eram do sexo masculino. A média de idade foi de 66 anos. Houve uma predominância de indivíduos procedentes do interior do estado de Sergipe, casados ou em união estável, católicos, de baixa escolaridade, aposentados e com renda menor ou igual a um salário mínimo ( Tabela 1 ). 

**Tabela 1:** Características socioeconômicas.

**Variáveis**	**n** (%)
**Sexo**	
Feminino	58 (45,7%)
Masculino	69 (54,3%)
**Procedência**	
Capital	44 (34,6%)
Interior	83 (65,4%)
**Estado civil**	
Com companheiro(a)	65 (51,2%)
Sem companheiro(a)	62 (48,8%)
**Afiliação religiosa**	
Católico	110 (86,6%)
Evangélico	17 (13,4%)
**Escolaridade**	
Analfabeto	33 (26%)
Fundamental – 1º grau	81 (63,8%)
Ensino médio – 2º grau ou mais	13 (10,2%)
**Estado ocupacional**	
Empregado	25 (19,7%)
Aposentado	102 (80,3%)
**Renda familiar**	
De 0 a 1 SM	93 (73,2%)
2 ou mais SM	34 (26,8%)

SM: salário mínimo.

 A maior parte da amostra, 101 indivíduos (79,5%), referiu ter ido às consultas com médico do Programa de Saúde da Família (PSF) nos últimos 12 meses. Noventa e cinco (74,8%) dos entrevistados relataram que não eram tabagistas e nem etilistas. Além disso, 92 (72,4%) dos entrevistados já tinham sido submetidos a cirurgias como – colecistectomia, herniorrafia inguinal, histerectomia, procedimentos para tratamento de varizes de membros inferiores (MMII), vasectomia, prostatectomia, cirurgia de catarata, e 59 (46,5%) sofreram amputação de MMII. A maioria dos participantes também relatou dor em MMII, e 77 (60,6%) não conseguiam deambular. 

 Entre as comorbidades associadas no grupo estudado prevaleceram a hipertensão arterial sistêmica (HAS) e a DM. A Tabela 2 apresenta a frequência dessas comorbidades na amostra, bem como os medicamentos utilizados para seu o tratamento. 

**Tabela 2:** Comorbidades associadas prevalentes e medicamentos mais utilizados.

**Comorbidades**	**n** (%)
**Hipertensão arterial sistêmica**	99 (78,0%)
Inibidores da enzima conversora da angiotensina	53 (41,7%)
Bloqueadores do receptor da angiotensina II	58 (45,7%)
Diurético	18 (14,2%)
Bloqueador de canais de cálcio	10 (7,9%)
Betabloqueador	9 (7,1%)
Alfa 2-agonista	3 (2,4%)
**Diabetes melito**	27 (21,4%)
Metformina	42 (33,1%)
Sulfoniureia	51 (40,2%)
Insulina NPH	3 (2,4%)
Inibidor de DPP4 (vildagliptina)	3 (3,1%)

 Em uma média geral, os menores escores foram obtidos no domínio físico, domínio meio ambiente e em QV total, com, respectivamente, 64,2±27,9, 97,2±20,3 e 97,9±20,4 pontos, enquanto o maior escore foi no domínio de relações sociais, com média de 150,2±28,9 pontos. Além disso, foi encontrada uma variação significativa nos domínios físico (p = 0,024), psicológico (p = 0,015), meio ambiente (p = 0,020) e qualidade de vida total (p = 0,039), como demonstrado na Tabela 3 . 

**Tabela 3:** Escores de QV.

**Domínios de QV**	**Total** **Média±DP**	**Homens** **Média±DP**	**Mulheres** **Média±DP**	**p-valor**
Físico	64,2±27,9	35,0±15,3	29,2±12,6	0,024
Psicológico	113,2±27,4	59,8±13,5	53,4±13,9	0,015
Relações sociais	150,2±28,9	74,9±15,3	75,3±13,6	0,873
Meio ambiente	97,2±20,3	50,8±10,8	46,4±9,5	0,020
QV total	97,9±20,4	51,1±10,3	46,8±10,1	0,039

DP: desvio padrão; QV: qualidade de vida.

 Na análise intragrupo da QV, os homens obtiveram uma pontuação média significativamente maior em todos os domínios quando comparados às mulheres (p = 0,098), exceto para o domínio relações sociais, em que as mulheres obtiveram uma pontuação maior (p = 0,873). 

 Em relação às variáveis sociodemográficas, os indivíduos com maior nível de escolaridade (ensino médio ou mais) e empregados obtiveram escores de QV significativamente maiores em todos os domínios, exceto no de relações sociais, quando comparados aos de baixo nível escolar (p = 0,26) e desempregados (p < 0,05). 

 Os pacientes que relataram realizar atividade física regularmente e sentir dor nos MMII apresentaram melhores médias de QV nos domínios físico (p = 0,27) e meio ambiente (p = 0,28), exceto no de relações sociais, quando comparados àqueles que não realizavam atividades físicas. Além disso, os entrevistados que relataram impedimento para caminhar devido à dor em MMII obtiveram escores menores em todos os domínios (p = 0,01). Vale ressaltar que a presença de feridas influenciou negativamente os domínios físico (p = 0,011), psicológico (p = 0,013) e QV total (p = 0,003). 

 Na análise das comorbidades associadas mais prevalentes, apenas a HAS influenciou negativamente a QV nos domínios psicológico, meio ambiente e QV total (p = 0,009; p = 0,022; p = 0,008, respectivamente). Com relação ao tipo de medicamento usado, não foi possível verificar interferência na QV, devido à grande variedade de associações. 

## DISCUSSÃO

 O aumento do número de indivíduos com DCNT, em especial DAP, tem sido uma preocupação para o Ministério da Saúde em todo país 5 . Essa é a realidade de muitos indivíduos, o que tem levado à constatação da ocorrência de baixos escores dos domínios de QV em seus portadores, exceto o de relações sociais, conforme o encontrado no presente estudo. 

 Os presentes achados demonstraram que os indivíduos com DAP do sexo masculino e maiores níveis de escolaridade apresentam melhor QV. Esses achados estão de acordo com os descritos por Carvalho et al. 11 quando estudaram a influência da HAS na QV. 

 Com relação ao sexo, Ahluwalia et al. 12 ^,^ 13 e Nogales 14 afirmaram que a menor pontuação de QV das mulheres em relação aos homens possivelmente esteja associada a fatores como diferenças biológicas e novos papéis na sociedade, bem como ao aumento da expectativa de vida. Em relação à escolaridade, de acordo com Saraiva et al. 15 , o maior nível de compreensão das informações pode possibilitar um melhor conhecimento da doença e, consequentemente, uma maior adesão ao tratamento. 

 Oza et al. 4 utilizaram o WHOQOL-Bref e MINICHAL como instrumento para avaliação da QV. Os autores encontraram diferenças em relação à média dos escores entre os domínios do WHOQOL-Bref e constatarem que o domínio de relações sociais apresentou um escore menor, enquanto a pontuação do domínio físico foi o mais alto. 

 Çeviker et al. 16 também utilizaram o WHOQOL-Bref, e demonstraram que pacientes com doenças crônicas venosas tiveram valores piores na QV em comparação aos controles e que, em uma análise univariada, todos os domínios (físico, psicológico, relações sociais e meio ambiente) foram afetados. Esses achados foram parcialmente similares aos encontrados no presente estudo. 

 Malaquias et al. 17 , Evangelista et al. 18 e Costa et al. 19 relataram que uma doença vascular, a úlcera venosa, pode levar a um isolamento social, constrangimento, sentimento de discriminação pela sociedade e pela família, explicando o fato de o aspecto psicológico ter baixa pontuação, corroborando com a baixa pontuação do domínio psicológico na presença de feridas. 

 Segundo Arslantas et al. 20 , que estudaram a HAS, a falta de consistência dos estudos acerca de QV pode ser justificada pelas diferenças entre as populações escolhidas para esse tipo de estudo, pelas diferentes características sociodemográficas e pela gravidade das doenças e comorbidades associadas. 

## CONCLUSÃO

 Neste estudo, verificou-se uma maior pontuação no domínio de relações sociais e uma menor pontuação nos domínios meio ambiente e QV total. Por meio da comparação entre os sexos, foi possível observar que as mulheres apresentam uma menor pontuação em todos os domínios, exceto no de relações sociais, quando comparadas aos homens. 

 Diante desses resultados, o presente estudo pode proporcionar melhor conhecimento e compreensão sobre o impacto das doenças vasculares na QV. Assim, é necessário que os indivíduos portadores dessa classe de doença, os profissionais de saúde e as organizações sociais e familiares cooperem para o enfrentamento dos fatores que influenciam negativamente a QV dessas pessoas.
